# Association between chronic obstructive pulmonary disease and cardiovascular disease in adults aged 40 years and above: data from NHANES 2013–2018

**DOI:** 10.1186/s12890-023-02606-1

**Published:** 2023-08-31

**Authors:** Hong Chen, Xiaojia Luo, Yuejun Du, Chenyun He, Yanjun Lu, Zixuan Shi, Jin Zhou

**Affiliations:** 1 Department of Respiratory and Critical Care Medicine, Chengdu Second People’s Hospital, Chengdu, China; 2 Department of Cardiovascular Medicine, Chengdu Second People’s Hospital, Chengdu, China; 3 Department of Emergency, Chengdu Second People’s Hospital, Chengdu, China; 4 Department of Medical Oncology, Sichuan Clinical Research Center for Cancer, Sichuan Cancer Hospital and Institute, Sichuan Cancer Center, Affiliated Cancer Hospital of University of Electronic Science and Technology of China, Chengdu, China

**Keywords:** Chronic obstructive pulmonary disease (COPD), Cardiovascular disease (CVD), NHANES, Aging

## Abstract

**Background:**

Chronic obstructive pulmonary disease (COPD) and cardiovascular disease (CVD) are two major age-related diseases prevalent in the elderly. However, it is unclear whether there is a higher prevalence of one or more CVDs in COPD patients compared to those without COPD, and the magnitude of this increased prevalence.

**Methods:**

This population-based cross-sectional study was conducted using data from the National Health and Nutrition Examination Survey (NHANES) 2013–2018 among American adults aged 40 years and above. Multivariable logistic regression models (including unadjusted model, minimally adjusted model, and fully adjusted model) were conducted to investigate the association between COPD and the prevalence of one or more CVDs, including coronary heart disease, heart failure, angina pectoris, heart attack, diabetes, and stroke.

**Results:**

This study included 11,425 participants, consisting of 661 participants with COPD and 10,764 participants without COPD. COPD patients had a significantly higher prevalence of CVD than those without COPD (59.6% vs. 28.4%). After adjusting for covariates, COPD was significantly associated with the prevalence of one CVD (OR = 2.2, 95% CI = 1.6–3.0, p < 0.001), two or more CVDs (OR = 3.3, 95% CI = 2.2–5.0, p < 0.001), and three or more CVDs (OR = 4.3, 95% CI = 2.9–6.5, p < 0.001).

**Conclusions:**

Patients with COPD have a higher prevalence of one or more CVDs compared with those without COPD. Our findings highlight the importance of CVD prevention and management in patients with COPD.

## Introduction

Chronic obstructive pulmonary disease (COPD) and cardiovascular disease (CVD) are two major age-related diseases that are prevalent in the elderly population. COPD, a common respiratory disorder characterized by restricted airflow, has a morbidity of approximately 10% in adults aged 40 and above, increasing with age. Currently, COPD is the third-leading cause of death worldwide [1–3]. CVD, on the other hand, is a term used to refer to a range of disorders involving the heart or blood vessels, including coronary heart disease (CHD), heart failure (HF), stroke, arrhythmias, etc. CVD is a serious health issue that leads to high mortality and morbidity, with more than 17.3 million deaths worldwide attributed to CVD annually [4–7].

Evidence suggested that CVD is the most common comorbidity in COPD patients, and the association between COPD and CVD is intricate. Apart from shared risk factors such as smoking and aging, inflammation might be one of the common mechanisms that link these disorders [8–10]. Furthermore, the presence and progression of CVD are among the triggering factors of exacerbations in COPD patients, while exacerbations in COPD patients can also trigger adverse cardiovascular events [1, 11]. However, it is unclear whether there is a higher prevalence of one or more CVDs in COPD patients compared to those without COPD, and the magnitude of this increased prevalence.

Therefore, we conducted a population-based cross-sectional study using data from the National Health and Nutrition Examination Survey (NHANES) to investigate the association of COPD with CVD prevalence in American adults aged 40 and above, specifically with the prevalence of one or more CVDs and specific CVD.

## Methods

### Study population and data source

**Fig. 1:**
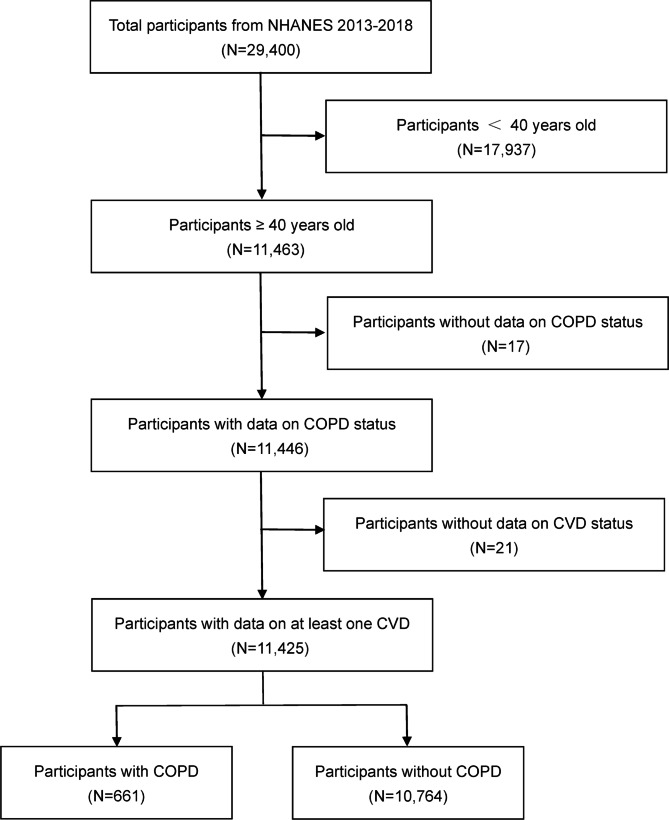
Flow chart of patient selection from the NHANES 2013–2018. NHANES, National Health and Nutrition Examination Survey; COPD, chronic obstructive pulmonary disease; CVD, cardiovascular disease

In this study, we utilized data from three cycles of the NHANES survey (NHANES 2013–2018), which provides the latest available data. We did not include the 2017-March 2020 Pre-Pandemic data, since only a portion of the required variables for the study were available. NHANES is a cross-sectional population-based survey aimed at collecting information on the health and nutritional status of adults and children in the United States (http://www.cdc.gov/nchs) provided by the National Center for Health Statistics (NCHS). The survey uniquely combines interviews and physical examinations. The results are used to determine the prevalence of major diseases and relevant factors and to provide the foundation for national standards on height, weight, and blood pressure. A total of 29,400 participants were investigated for their health status in three cycles. After excluding participants under the age of 40 and those missing information on the presence of COPD or CVD, a total of 11,425 participants were ultimately included in our analysis, including 661 participants with COPD and 10,764 participants without COPD **(**Fig. 1**)**. The survey protocol was approved by the Institutional Review Board of NCHS and all participants provided written informed consent.

### Exposure variable

Whether a participant had COPD was included in this study as the exposure variable. The values of this variable were extracted from NHANES questionnaire data. In the Medical Conditions section (MCQ160o), investigators asked participants whether a doctor had informed them that they had COPD. Participants who answered “yes” were included in the COPD group, while those who answered “no” were included in the control group. Participants who had missing data for this question were excluded from this study.

### Outcome variables

The outcome variable in this study is whether a participant had any of the common CVDs, including coronary heart disease, heart failure, angina, heart attack, diabetes, and stroke. The values of these variables were obtained from NHANES questionnaire data (Medical Conditions section: coronary heart disease [MCQ160c], heart failure [MCQ160b], angina pectoris [MCQ160d], heart attack [MCQ160e], diabetes [DIQ010], and stroke [MCQ160f]). Investigators asked participants whether a doctor had informed them that they had any of these CVDs, and participants who provided a clear answer of “yes” or “no” for any of these CVDs were considered to have the respective condition. Participants who did not provide any information about CVDs were excluded from this study.

### Covariates

This study included four categories of covariates for analysis, including baseline demographic characteristics data of participants (age, gender, body mass index [BMI], and ethnicity), lifestyle (smoking status, education level, and annual family income), inflammation indicators (blood eosinophil counts), and comorbidities associated with COPD (chronic bronchitis, emphysema, and asthma). These variables were obtained from the demographics data, examination data, laboratory data, and questionnaire data of NHANES, and their corresponding measurement methodologies are available on the NHANES website.

### Statistical analysis

All analyses were conducted with the EmpowerStats software (version 4.1), Stata software (version 15), and R package (version 4.0.4). We utilized multivariable logistic regression models to investigate the association between COPD and the prevalence of CVD and presented the results as odds ratios (ORs) with corresponding 95% confidence intervals (CIs). In accordance with the STrengthening the Reporting of OBservational studies in Epidemiology (STROBE) statement [12], we conducted three models to exclude the effects of covariates and determine whether COPD is significantly associated with a higher prevalence of CVD: unadjusted model, with no covariate adjustment (unadjusted OR); minimally adjusted model, with adjustment of age, gender, and race (minimally adjusted OR); and fully adjusted model, with adjustment of age, gender, race, body mass index, smoking status, education level, annual family income, blood eosinophil counts, and comorbidities, including asthma, chronic bronchitis, and emphysema (fully adjusted OR). We implemented an analysis procedure to account for the intricate and multistage survey design. To adjust for the impact of weight, strata, and clusters, we incorporated three vital variables from the demographic dataset of NHANES, including full sample 6 year MEC exam weight (WTMEC6YR), masked variance pseudo-stratum, and masked variance pseudo-PSU. Since three cycles of NHANES data were analyzed, WTMEC6YR was calculated as WTMEC6YR = 1/3 × WTMEC2YR according to the NHANES recommendation. Statistical significance was determined at a two-sided *p-*value of less than 0.05.

The primary outcome of this study was to investigate the association between COPD and the prevalence of one type of common CVD (participants had at least one CVD, including coronary heart disease, heart failure, angina pectoris, heart attack, diabetes, and stroke). The subgroup analyses included (1) the prevalence of two or more CVDs and three or more CVDs in participants with COPD compared to participants without COPD. (2) The prevalence of specific CVD in participants with COPD compared to participants without COPD. A forest plot was included to show the CVD prevalence of COPD patients compared to participants without COPD.

## Results

### Study population

A total of 11,425 participants aged 40 years or above were included in this study. The COPD group consisted of 661 participants, while the control group had 10,764 participants. The two groups exhibited significant differences in baseline demographic characteristics, lifestyle, inflammation indicators, and comorbidities (Table 1). Compared to the control group without COPD, participants with COPD were older, with a population aged 65 and above, current smokers, Non-Hispanic Whites, increased blood eosinophil counts (≥ 300/µL), and lower annual family income accounting for a larger proportion, while those with higher education levels accounted for a smaller proportion. In addition, compared with participants without COPD, participants with COPD had a higher prevalence of CVD with a larger proportion of individuals with one or more CVDs.

**Table 1:** Characteristics of study participants from NHANES 2013–2018 stratified by COPD status

Characteristic	COPD (N = 661)	Control (N = 10,764)	*p*
Age (years)	64.3 ± 10.2	58.1 ± 11.7	< 0.001
Age (%)			< 0.001
＜65 years old	54.5	69.8	
≥ 65 years old	45.5	30.2	
Gender (%)			0.8290
Male	47.6	47.2	
Female	52.4	52.8	
BMI (kg/m^2^)	30.0 ± 8.1	29.8 ± 6.7	0.3203
Smoking status (%)			< 0.001
Current	46.4	66.2	
Never	53.6	33.8	
Race (%)			< 0.001
Mexican American	1.7	7.3	
Other Hispanic	2.1	5.4	
Non-Hispanic White	80.5	68.0	
Non-Hispanic Black	7.2	10.8	
Other Race	8.5	8.6	
Blood eosinophil counts (%)			< 0.001
< 300/µL	66.8	74.8	
≥ 300/µL	33.2	25.2	
Annual family income (USD)			< 0.001
< 35,000	58.3	28.0	
≥ 35,000 and < 100,000	33.4	41.9	
≥ 100,000	8.3	30.0	
Education level (%)			< 0.001
< High school	21.4	13.8	
High school	34.1	22.5	
＞High school	44.5	63.7	
Asthma (%)	41.0	12.5	< 0.001
chronic bronchitis (%)	42.1	5.4	< 0.001
Emphysema (%)	35.8	0.6	< 0.001
Have one CVD (%)	57.8	22.5	< 0.001
Have two or more CVDs (%)	31.9	6.3	< 0.001
Have three or more CVDs (%)	18.3	2.7	< 0.001
Coronary heart disease (%)	23.7	4.7	< 0.001
Heart failure (%)	18.9	2.8	< 0.001
Angina pectoris (%)	15.8	2.6	< 0.001
Heart attack (%)	21.9	4.3	< 0.001
Diabetes (%)	29.6	15.0	< 0.001
Stroke (%)	14.1	3.9	< 0.001

Mean ± SD for continuous variables, p-value was calculated by weighted linear regression model; % for categorical variables, p-value was calculated by weighted chi-square test

*Abbreviations: NHANES* National Health and Nutrition Examination Survey, *COPD* Chronic obstructive pulmonary disease, BMI, Body mass index, *CVD* Cardiovascular disease

### Association between COPD and prevalence of one or more CVDs

**Fig. 2:**
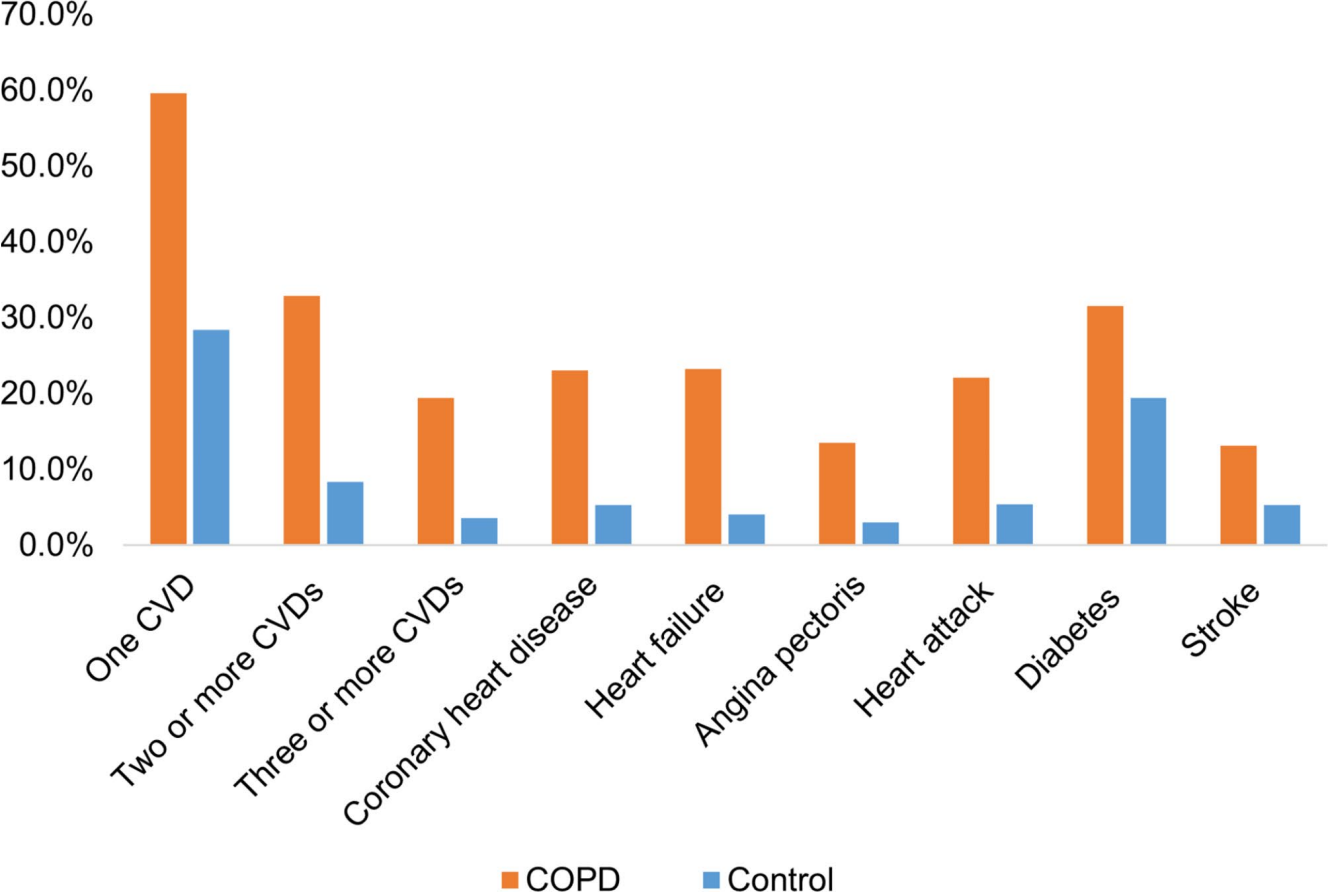
Prevalence of CVD in COPD and control groups. COPD, chronic obstructive pulmonary disease; CVD, cardiovascular disease

**Fig. 3:**
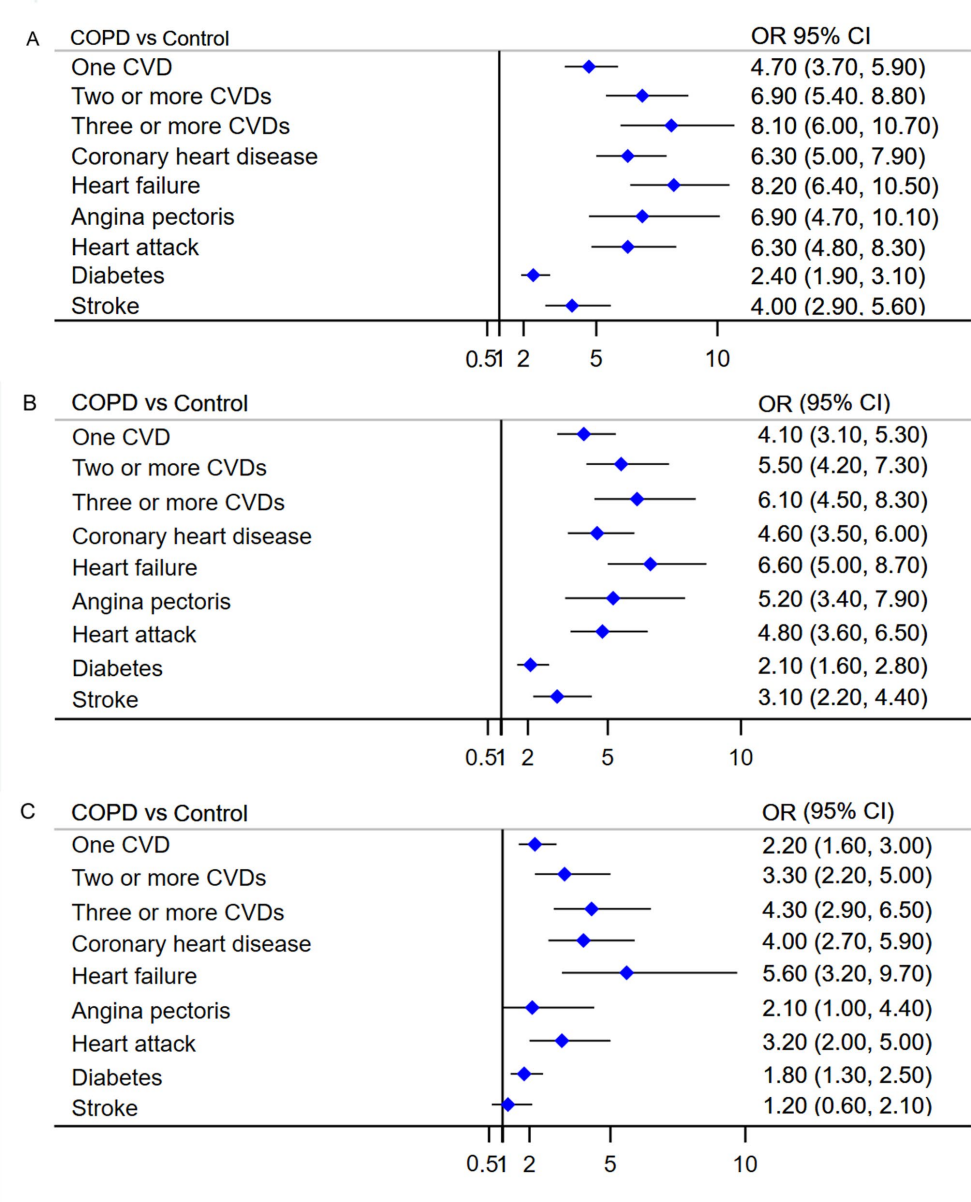
Association between COPD and prevalence of CVD in adults aged 40 years and above. **A**, Unadjusted model: with no covariate adjustment; **B**, Minimally adjusted model: with adjustment of age, gender, and race; **C**, Fully adjusted model: with adjustment of age, gender, race, body mass index, smoking status, education level, annual family income, blood eosinophil counts, and comorbidities, including asthma, chronic bronchitis, and emphysema. COPD, chronic obstructive pulmonary disease; CVD, cardiovascular disease; OR, odds ratio; CI, confidence interval

The overall prevalence of one CVD, two or more CVDs, and three or more CVDs in the COPD group were 59.6%, 32.8%, and 19.4%, respectively, whereas the prevalence in the control group were 28.4%, 8.3%, and 3.5%, respectively (Fig. 2). The multivariable logistic regression models for all three models showed a significant association between COPD and a higher prevalence of one or more CVDs, and participants with COPD were more likely to have more CVDs than those without COPD. In the unadjusted model, participants with COPD tended to have a higher prevalence of one, two or more CVDs, and three or more CVDs (one CVD: OR = 4.7, 95% CI = 3.7–5.9, p < 0.001; two or more CVDs: OR = 6.9, 95% CI = 5.4–8.8, p < 0.001; three or more CVDs: OR = 8.1, 95% CI = 6.0–10.7, p < 0.001). After adjusting for the effects of covariates, or confounders, this association still existed in the minimally adjusted model (one CVD: OR = 4.1, 95% CI = 3.1–5.3, p < 0.001; two or more CVDs: OR = 5.5, 95% CI = 4.2–7.3, p < 0.001; three or more CVDs: OR = 6.1, 95% CI = 4.5–8.3, p < 0.001) and the fully adjusted model (one CVD: OR = 2.2, 95% CI = 1.6–3.0, p < 0.001; two or more CVDs: OR = 3.3, 95% CI = 2.2–5.0, p < 0.001; three or more CVDs: OR = 4.3, 95% CI = 2.9–6.5, p < 0.001) (Fig. 3). These results are summarized in Table 2.

**Table 2:** Comparison of CVD prevalence in COPD and control groups

CVD prevalence	Unadjusted OR (95% CI), *p*	Minimally adjusted OR (95% CI), *p*	Fully adjusted OR (95% CI), *p*
One CVD			
Control	Reference	Reference	Reference
COPD	4.7 (3.7–5.9) < 0.001	4.1 (3.1–5.3) < 0.001	2.2 (1.6–3.0) < 0.001
Two or more CVDs			
Control	Reference	Reference	Reference
COPD	6.9 (5.4–8.8) < 0.001	5.5 (4.2–7.3) < 0.001	3.3 (2.2–5.0) < 0.001
Three or more CVDs			
Control	Reference	Reference	Reference
COPD	8.1 (6.0–10.7) < 0.001	6.1 (4.5–8.3) < 0.001	4.3 (2.9–6.5) < 0.001
Coronary heart disease			
Control	Reference	Reference	Reference
COPD	6.3 (5.0–7.9) < 0.001	4.6 (3.5–6.0) < 0.001	4.0 (2.7–5.9) < 0.001
Heart failure			
Control	Reference	Reference	Reference
COPD	8.2 (6.4–10.5) < 0.001	6.6 (5.0–8.7) < 0.001	5.6 (3.2–9.7) < 0.001
Angina pectoris			
Control	Reference	Reference	Reference
COPD	6.9 (4.7–10.1) < 0.001	5.2 (3.4–7.9) < 0.001	2.1 (1.0–4.4) 0.060
Heart attack			
Control	Reference	Reference	Reference
COPD	6.3 (4.8–8.3) < 0.001	4.8 (3.6–6.5) < 0.001	3.2 (2.0–5.0) < 0.001
Diabetes			
Control	Reference	Reference	Reference
COPD	2.4 (1.9–3.1) < 0.001	2.1 (1.6–2.8) < 0.001	1.8 (1.3–2.5) 0.003
Stroke			
Control	Reference	Reference	Reference
COPD	4.0 (2.9–5.6) < 0.001	3.1 (2.2–4.4) < 0.001	1.2 (0.6–2.1) < 0. 624

*Abbreviations: CVD* Cardiovascular disease, *COPD* Chronic obstructive pulmonary disease, OR Odds ratio, CI confidence interval

Unadjusted OR: with no covariate adjustment. Minimally adjusted OR: with adjustment of age, gender, and race. Fully adjusted OR: with adjustment of age, gender, race, body mass index, smoking status, education level, annual family income, blood eosinophil counts, and comorbidities, including asthma, chronic bronchitis, and emphysema

### Association between COPD and specific CVDs

The prevalence of coronary heart disease, heart failure, angina pectoris, heart attack, diabetes, and stroke were 23.7%, 18.9%, 15.8%, 21.9%, 29.6%, 14.1% in the COPD group, and 4.7%, 2.8%, 2.6%, 4.3%, 15.0%, 3.9% in the control group, respectively (Fig. 2). The multivariable logistic regression models for all three models showed a significant association between COPD and the prevalence of four specific CVDs (coronary heart disease, heart failure, heart attack, and diabetes). In the unadjusted models, COPD was significantly associated with a higher prevalence of all 6 specific CVDs, but after adjusting for confounding factors, COPD was not significantly associated with stroke and angina pectoris (Fig. 3). These results are summarized in Table 2.

## Discussion

In this study, we utilized population-based cross-sectional data from the 2013–2018 NHANES to investigate the association between COPD and CVD prevalence among American adults aged 40 years and above. Our results suggested that COPD patients had a significantly higher prevalence of CVD than those without COPD. After adjusting for covariates, COPD was significantly associated with a higher prevalence of one or more CVDs. Compared to participants without COPD, the prevalence of one, two or more, or three or more CVDs increased successively in COPD patients. In addition, after adjusting for potential confounding factors, COPD was significantly associated with a higher prevalence of several specific CVDs, including coronary heart disease, congestive heart failure, heart attack, and diabetes. Overall, these findings suggested that COPD is associated with a higher prevalence of CVD. To our knowledge, our study revealed, for the first time, a gradual increase in the prevalence of concomitant more CVDs in COPD patients as compared to individuals without COPD. Our findings provided important insights into the management of COPD and CVD and therefore had important implications for clinical practice.

COPD is currently a major cause of morbidity and mortality worldwide, particularly in individuals over 40 years of age [1, 13, 14]. As the disease progresses, patients experience recurrent exacerbations, continuous decline in lung function, and progressive reductions in quality of life and physical activity, ultimately leading to death [15, 16]. The natural course and prognosis of COPD are influenced by various clinical factors, with comorbidities being an important consideration [17–19]. CVD is the most common and prevalent comorbidity in COPD patients, and it is also a major cause of mortality in this population. Even in moderate COPD, exacerbations of CVD lead to a significant proportion of death [8, 20, 21]. COPD and CVD commonly coexist and interact with each other. The presence and worsening of CVD are one of the triggering factors for exacerbations of COPD, while exacerbations of COPD can trigger malignant cardiovascular events, even resulting in cardiovascular death. Several studies found that the prevalence of myocardial infarction increased by twofold within five days after an exacerbation in COPD patients, and the prevalence of stroke increased by 40% within ten days [1, 11, 22]. Therefore, it is essential to find effective treatment strategies that can reduce both COPD and CVD comorbidities to improve the prognosis of COPD patients and reduce their cardiovascular mortality.

Our research supported previous studies that found a higher prevalence of CVD in COPD patients than in those without COPD and provided additional information on this association. A study using data from a large UK database of over 1.2 million patients aged 35 and above identified around 30,000 patients with COPD, who were around five times more likely to have CVD than those without COPD [22]. In another study of 351 patients with end-stage COPD, 60% had clinically significant coronary artery disease on angiography, including 53% occult [23]. A meta-analysis showed that compared with individuals without COPD, COPD patients were more likely to be diagnosed with CVD (OR 2.46, 95% CI 2.02-3.00) [24]. In this study, in terms of the prevalence of specific CVD in COPD patients, the order from high to low is as follows: diabetes, coronary heart disease, heart attack, heart failure, angina pectoris, and stroke. Our study supported the above findings with the use of large sample size and nationally representative data from NHANES 2013–2018 and found that in the US population aged 40 and above with COPD, even after adjusting for the effects of relevant covariates, including baseline demographic characteristics, lifestyle, inflammation indicators, and comorbidities, the prevalence of various common CVDs was significantly higher than in the population without COPD. These findings indicated that COPD is significantly associated with a higher prevalence of some CVDs, including coronary heart disease, heart failure, heart attack, and diabetes. Our results suggested that compared to participants without COPD, the prevalence of stroke in COPD patients was higher (14.1% vs. 3.9%), which was consistent with previous studies conducted by Corlateanu et al. [25] and Kim et al. [26]. The study conducted by Corlateanu et al. also suggested that due to the presence of various comorbidities related to COPD, it is challenging to determine whether COPD acts as an independent risk factor for stroke or if it is influenced by confounding effects [25]. A study conducted by O’Donnell et al. found that one major risk factor for stroke is smoking [27]. Smoking is currently recognized as the most important risk factor for COPD. In a study conducted by Finkelstein et al. [28], COPD is significantly associated with a higher prevalence of stroke. However, in this study, after adjusting for multiple potential confounding variables, COPD is not significantly associated with stroke prevalence. Further studies are needed to elucidate the association between COPD and stroke and whether there is a causal relationship between the two. Furthermore, our results suggested that compared to participants without COPD, the trend of increased prevalence for one, two or more CVDs, and three or more CVDs is more pronounced in COPD patients. This trend further indicated that COPD is significantly associated with a higher prevalence of stroke.

The potential mechanisms behind the coexistence of COPD and CVD remain unclear. The complex association between the two is not only due to their shared risk factors such as smoking, aging, diet, air pollution, and a sedentary lifestyle but also the common mechanisms of pathogenesis, including inflammation, hypoxia, oxidative stress, etc. [29–32]. Smoking can induce a variety of inflammatory responses in susceptible individuals, and this chronic systemic inflammatory response is not only related to COPD, but also closely linked with the formation, progress, and rupture of atherosclerotic plaque, and can further induce coronary heart disease, heart failure, and even cardiovascular death [33, 34]. Hypoxia in COPD patients is also involved in the development of CVD. Hypoxia can affect atherosclerosis progression in various ways, including inflammatory responses, oxidative stress, upregulation of cell surface adhesion molecules, changes in hemodynamics, and thrombosis. Furthermore, hypoxia can also change cardiac output and blood pressure, and affect vascular function [33, 35]. In addition, oxidative stress is one of the typical pathological changes of COPD patients and plays an important role in the morbidity, development, and rupture of atherosclerosis. Oxidative stress first causes endothelial cell damage and thus initiates atherosclerosis, while the increase of Reactive Oxygen Species (ROS) will induce the expression of sensitive transcription factors and their downstream genes to be up-regulated, which then intensifies the inflammatory response, which in turn intensifies oxidative stress [33]. Moreover, exacerbations can worsen tachycardia, elevated pulmonary artery pressure, arterial systolic dysfunction, and hemodynamic disorders, and lead to acute cardiac dysfunction in COPD patients with CVD [36, 37].

The main advantage of our study is that we used data from NHANES 2013–2018. NHANES adopts standardized participant information collection with a large sample size, which can provide nationally representative sample information. Therefore, the present study has high applicability. In addition, we analyzed the association between COPD and CVD from multiple dimensions, including the association of COPD with the prevalence of one CVD, two or more CVDs, and three or more CVDs, and the association of COPD with the prevalence of a specific CVD. Our study has some limitations. First, the data used in this study were obtained from an online database, and as a cross-sectional study, our findings can only demonstrate the existence of an association between COPD and CVD, rather than a causal relationship between the two. Second, the severity of COPD, such as lung function, disease duration, and history of exacerbations was not included in the NHANES dataset. Therefore, it is unclear whether the observed association exists and to what extent it does so in different severity levels of COPD. Third, the diagnosis of COPD and CVD was based on questionnaire data obtained from study participants and therefore medical misdiagnosis errors occurred when a healthcare professional failed to correctly identify these conditions. This may introduce bias to our results. It highlights the importance of education and training for healthcare professionals and participants of NHANES to obtain accurate information on the diseases of the participants. Further prospective studies are needed to confirm the association between COPD and CVD and whether there is causality between the two. Finally, it should be noted that participants in the NHANES study were predominantly Non-Hispanic White and Non-Hispanic Black individuals from the United States, and thus generalization of our findings to other populations should be approached with caution.

## Conclusion

Patients with COPD have a higher prevalence of one or more CVDs (including coronary heart disease, heart failure, heart attack, and diabetes) compared with those without COPD. Our findings highlight the importance of CVD prevention and management in patients with COPD.

## Data Availability

The data analyzed in this study are from the NHANES 2013–2018, which are publicly available and can be downloaded from the NHANES website: http://www.cdc.gov/nchs/nhanes.htm.

## Abbreviations

COPD: Chronic obstructive pulmonary disease
CVD: Cardiovascular disease
NHANES: National Health and Nutrition Examination Survey
NCHS: National Centers for Health Statistics
CHD: Coronary heart disease
HF: Heart failure
OR: Odds ratio
CI: Confidence interval
